# Compressive Fatigue Behaviour of High-Strength Concrete and Mortar: Experimental Investigations and Computational Modelling

**DOI:** 10.3390/ma15010319

**Published:** 2022-01-03

**Authors:** Nadja Oneschkow, Tim Timmermann, Stefan Löhnert

**Affiliations:** 1 Institute of Building Materials Science, Leibniz University Hannover, Appelstraße 9a, 30167 Hannover, Germany; t.timmermann@baustoff.uni-hannover.de; 2 Institute of Mechanics and Shell Structures, Technische Universität Dresden, August-Bebel-Straße 30, 01219 Dresden, Germany; stefan.loehnert@tu-dresden.de

**Keywords:** high-strength concrete, high-strength mortar, acoustic emission, fatigue damage, computational modelling, gradient-enhanced damage

## Abstract

A high-strength concrete and mortar subjected to compressive fatigue loading were comparatively investigated using experimental and computational techniques. The focus of the investigations was on the influence of the coarse aggregate in high-strength concrete. Accordingly, the fatigue behaviour was analysed experimentally using the macroscopic damage indicators strain, stiffness and acoustic emission hits. The results clearly show differences in the fatigue behaviour between the concrete and the mortar, especially at the lower stress level investigated. The basalt coarse aggregate here improves the fatigue behaviour of the concrete. Indication of a negative effect can be seen at the higher stress level. A finite element approach with a gradient-enhanced equivalent strain-based damage model combined with a fatigue model was used for the computational simulation of the fatigue behaviour. The damage model includes a differentiation between tension and compression. The fatigue model follows the assumption of the reduction in the material strength based on the accumulated gradient-enhanced equivalent strains. A random distribution of spherically shaped basalt aggregates following a given particle size distribution curve is used for the simulation of concrete. The comparison of the experimentally and computationally determined strain developments of the concrete and mortar shows very good agreement.

## 1. Introduction

The application of concrete with increasingly higher compressive strengths enables the realisation of more slender concrete structures. Compared to massive structures, these slender structures are exposed to fatigue-relevant loads to a higher extent because of their lower ratio of deadweight to non-static loads. Moreover, special structures, such as wind energy plants or machine foundations, are generally exposed to fatigue loading with huge numbers of load cycles. Both the development of concrete types with increasingly higher compressive strengths and the expanded construction of wind energy plants have led to a growth of research activities in the field of the fatigue resistance of concrete in the last few decades. After focusing mainly on the number of cycles to failure, the latest research is especially focused on the concrete fatigue behaviour or rather damage development [1,2], which can be described by different damage indicators, such as strain, stiffness and, as an innovative experimental approach, acoustic emission (AE). However, only a little knowledge is currently available concerning the characteristics of the ongoing damage processes in concrete.

It is well-known that high-strength concrete has a denser binder matrix with increased compressive strength and a less pronounced and improved interfacial transition zone. Both properties lead to an improved microstructure. However, the influence of this improved microstructure and the presence of coarse aggregate on the fatigue behaviour under compressive fatigue loading has not yet been understood entirely.

Mehmel and Kern [3] investigated the fatigue behaviour of a normal strength concrete and cement stone comparatively. They defined the coarse aggregates as “solid phase” and the cement stone as “viscose phase”, and found that the interaction between these phases or, rather, the inhomogeneous stress distribution induced affects the fatigue behaviour of concrete to a high extent. Shah and Chandra [4] also observed that the interaction between the coarse aggregates and the cement stone is essential for the damage mechanisms under fatigue loading. The results of Thiele [2] for a normal strength concrete confirm the strong effect of the inhomogeneous three-dimensional stress distribution with a diffuse, spread compressive damaging effect in the cement matrix, and a localised, vertically orientated tensile damage. The big influence of the inhomogeneous stress distribution induced by coarse aggregates together with a strong stress level dependency of the compressive fatigue behaviour could be shown for high-strength concrete compositions in [5].

The modelling of concrete, including its complex material and damage behaviour, has reached a highly advanced state for monotonically increasing external loads. In the case of fatigue loading, the cyclic increase in damage, which additionally depends on, for example, the maximum and minimum stress level and the load frequency, has to be comprised by the computational model. Material models, such as a Drucker–Prager–Cap model [6] or microplane models [7], combined with classical damage approaches are, in most cases, unsuitable for fatigue simulations because damage generally reaches a state of saturation after a few cycles and further cycles do not lead to further damage and finally, fracture of the material. Consequently, during the last few decades, the mechanical and numerical modelling of fatigue has generally been based on phenomenological model assumptions, such as the well-known Paris law, which was used explicitly as part of the models. This means that a specific increase in damage or a particular crack increment prescribed by Paris law is assumed for every load cycle and the actual damage mechanisms in the background are not scrutinised. Such an approach, however, does not allow one to describe the fatigue damage behaviour in terms of a predictive model, which is applicable independently of the external load.

In addition to the phenomenological modelling of fatigue, a few damage models were developed especially for fatigue problems [8]. These models, however, may show weaknesses in non-cyclic applications. In the context of phase-field modelling, [9,10,11] recently proposed the idea of locally reducing the strength of the material depending on the local development of the strain and its accumulation. As a result, the entire strain history may have an influence on the remaining strength of the material. The results presented in [11] showed very good agreement with experimental observations not only for the Paris regime (phase II) but also for phase I and III of the well-known macroscopic s-shaped strain development [1,12]. In this paper, an extension of the approach presented in [11] is proposed regarding a damage model based on gradient-enhanced strains, where the reduction in the strength of the material is captured in a modification of the yield function for the critical gradient-enhanced equivalent strain.

The general challenge in developing and validating computational models is the limited knowledge regarding the important material behaviour patterns and their interpretation on a small length scale leading to the observable macroscopic material behaviour. This particularly concerns those models that can be applied to simulate the complex processes of fatigue damage accumulation in concrete’s microstructure. A close cooperation between researchers in computational mechanics and materials science is essential in this case.

A conjoint research project, which is part of the DFG Priority Programme SPP 2020 ‘Cyclic Deterioration of High-Performance Concrete in an Experimental-Virtual Lab’, investigated the compressive fatigue behaviour of high-strength concrete and mortar experimentally and numerically. In this paper, the numbers of cycles to failure and the damage indicators strain, stiffness and AE are analysed comparatively for a high-strength concrete and its corresponding mortar. Furthermore, a new modelling approach for the simulation of the concrete and mortar’s fatigue behaviour is described and the numerical results received are presented comparatively and discussed with the fatigue behaviour determined experimentally.

## 2. Experimental Programme

### 2.1. Concrete Composition and Specimen Preparation

The experimental investigations were conducted on a high-strength concrete RH1-B, which is the reference concrete composition in the research project (e.g., also [13,14]) and in the Priority Programme SPP 2020, and on the corresponding mortar M-B. The latter was obtained by sieving off the concrete components with grain sizes ≥ 2 mm. Nevertheless, the mortar contains a small volume of basalt with grain sizes < 2 mm. The composition of concrete RH1-B is given in Table 1. The water to cement (*w/c*) ratio is 0.35.

The 28-day compressive strength *f_cm,cube_* according to DIN EN 12390-3 [15] and the modulus of elasticity *E_cm_* according to DIN EN 12390-13 [16] were determined on three specimens each. The mean values are summarised in Table 2.

The compressive strength and the modulus of elasticity of the basalt coarse aggregate were determined using drill cores with a height of *h* = 300 mm and a diameter of *d* = 150 mm. The respective mean values of three tests each are *f_cm,b_* = 326 MPa and *E_m,b_* = 99,800 MPa.

Cylindrical specimens with a height of *h* = 180 mm and diameter of *d* = 60 mm were used in the fatigue tests. The concrete or mortar was filled into PVC formworks in two equal layers and each layer was mechanically compacted using a vibrating table. The PVC formwork of the concrete specimens was removed 7 days after concreting and the cylinders were stored in standard climate conditions (20 °C/65% R.H.) until testing. The treatment of the mortar specimens was different from that of the concrete specimens in order to avoid increased microcracking due to shrinkage. The mortar specimens were also stored in standard climate conditions, but the PVC formwork was removed 14 days after concreting. Subsequently, these specimens were stored wrapped in plastic foil for 14 days and then without foil for at least a further 28 days in standard climate conditions (gentle drying) until testing. The test surfaces of all specimens were plane-parallel ground and polished to achieve a more uniform stress distribution.

### 2.2. Test Programme and Experimental Set-Up

The fatigue tests were carried out force-controlled using a class 0.5 servo-hydraulic testing machine with a 500 kN actuator (according to ISO 7500-1 [17]). The full amplitude was applied in the first load cycle. The minimum stress level was kept constant at *S_min_* = 0.05 in all tests, while the maximum stress level was either *S_max_* = 0.85 or 0.70. The test frequency applied was *f_t_* = 1.0 Hz in all tests. The compressive fatigue reference strengths of the high-strength concrete and mortar were tested just before conducting the fatigue tests, using five specimens from the same batch having the same geometry as the specimens used in the fatigue tests. The compressive fatigue reference strengths for determining the axial test stresses based on the stress levels defined (*S_max_ = σ_max_/f_cm,ref_*; *S_min_ = σ_min_/f_cm,ref_*) were calculated as the mean values *f_cm,ref_* = 96 MPa for the concrete RH1-B and *f_cm,ref_* = 97 MPa for the mortar M-B. The age of specimens at fatigue testing was between 56 and 70 days.

**Figure 1:**
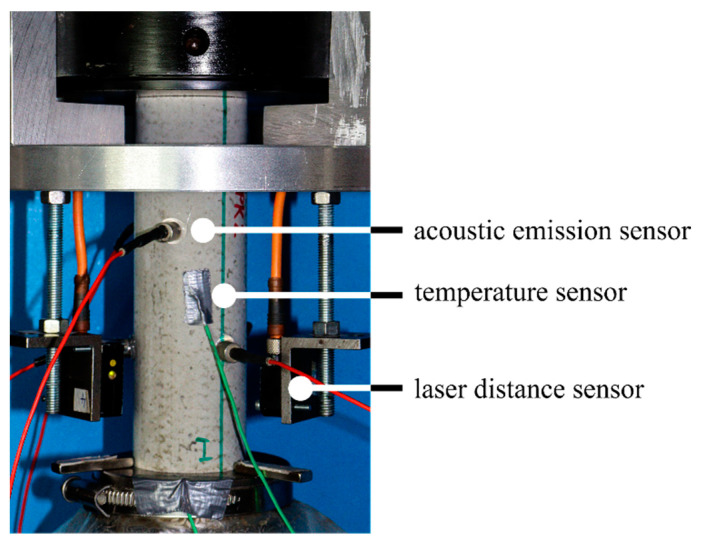
Experimental set-up [14].

The axial deformations were measured continuously in all fatigue tests using three laser distance sensors positioned on the circumference of the specimen at 0°, 120° and 240° (Figure 1). In addition, the axial force, the axial displacement of the actuator, the temperature of the specimen’s surface at mid-height and the ambient temperature were measured. The sampling rate was 300 Hz for all quantities measured. Furthermore, six AE sensors were attached to each specimen’s surface. The sensors had a wideband frequency response within the range of 250 to 1600 kHz, and were positioned at 60° from one another, alternating in the upper and lower third of the specimen. The AE hits are characterised as single transient signals. Based on pretests, a threshold of 40 dB was defined to separate the useable signal from the background noise.

### 2.3. Analysis of Damage Indicators

Fatigue damage indicators were used to describe the differences in the fatigue behaviour of the concrete and the mortar. The fatigue damage indicators: development of strain, stiffness and AE hits are analysed in this paper. These analyses include all cycles up to the last cycle before failure. This approach was established due to the general occurrence of fatigue failure at different stresses (but mostly in the range of the peak stresses) and the high-grade instable state of the microstructure, which is reached in the last cycle (cf. [1]).

The gradients of strain, stiffness and cumulated AE hits in phase II were determined between fixed values of related number of cycles *N/N_f_* = 0.20 and 0.80 in this paper for reasons of better comparability. The maximum and minimum peak strains for determining the strain developments were obtained by peak analyses of the sinusoidal strain curves determined by the three laser distance sensors. The three strain developments were subsequently averaged for each specimen. Concerning the mean strain development, a focus was set on the total growth of maximum and minimum strain and the gradient of strain development in phase II (cf. Equation (1)).

(1) $$grad\;\varepsilon_{min;max}^{0.2-0.8}=\frac{\sum_{i=1}^{0.2-0.8}\left(N_{i}-\bar{N}\right)\cdot \left(\varepsilon_{min;max,i}-{\bar{\varepsilon }}_{min;max}\right)}{\sum_{i=1}^{0.2-0.8}\left(N_{i}-\bar{N}\right)}$$

The stiffness was determined for each specimen as the secant modulus in the decreasing branch of the hysteresis loop (cf. Equation (2)). The gradient of stiffness development in phase II (i.e., reduction in stiffness per load cycle) was calculated equivalent to Equation (1). In addition, the percentile reduction in stiffness from the first to the last load cycle, with the initial value of stiffness in the first cycle as reference, was analysed.

(2) $$E_{S}=\frac{\sigma_{max}-\sigma_{min}}{\varepsilon_{max}-\varepsilon_{min}}$$

Regarding the analyses of the AE hits, they were summarised from cycle to cycle and displayed as developments of cumulated AE hits. The gradient of cumulated AE hits in phase II (i.e., increase in AE hits per load cycle) and the total number of AE hits in the complete fatigue process were used as parameters.

The mean values of the parameters of the damage indicators, calculated based on the single tests, are given in Table A2 in the appendix.

## 3. Experimental Results

### 3.1. Number of Cycles to Failure

**Figure 2:**
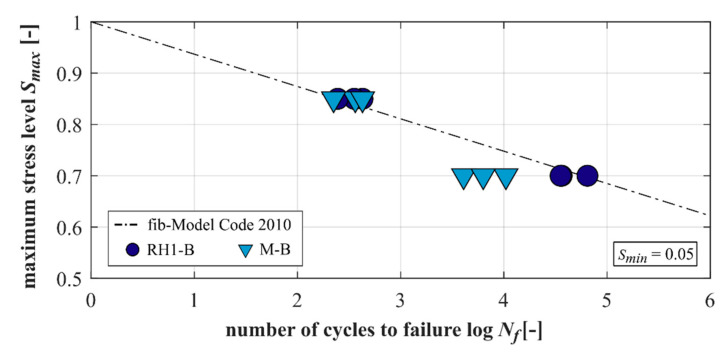
Numbers of cycles to failure for the concrete and the mortar.

It is worth mentioning that the increase in the specimen’s temperature due to mechanical loading was a maximal 14 K and, thus, relatively low. The correlation between the maximum compressive stress level *S_max_* and the numbers of cycles to failure log *N_f_* (single values) is shown in Figure 2 for the concrete and the mortar. Furthermore, the S–N curve for pure compressive fatigue loading of fib Model Code 2010 [18] is shown in Figure 2 for the purpose of comparison. Additionally, the single values and the mean values of the numbers of cycles to failure are given in Table A1.

The concrete RH1-B reached similar numbers of cycles to failure to its corresponding mortar M-B at the higher stress level *S_max_* = 0.85 and significantly higher numbers of cycles to failure at the lower stress level of *S_max_* = 0.70. This indicates, on the one hand, that no influence of the basalt coarse aggregate is recognisable for the higher stress level regarding the numbers of cycles to failure. At the lower stress level, on the other hand, the presence of the coarse aggregate leads to an improved fatigue behaviour.

### 3.2. Damage Indicators

**Figure 3:**
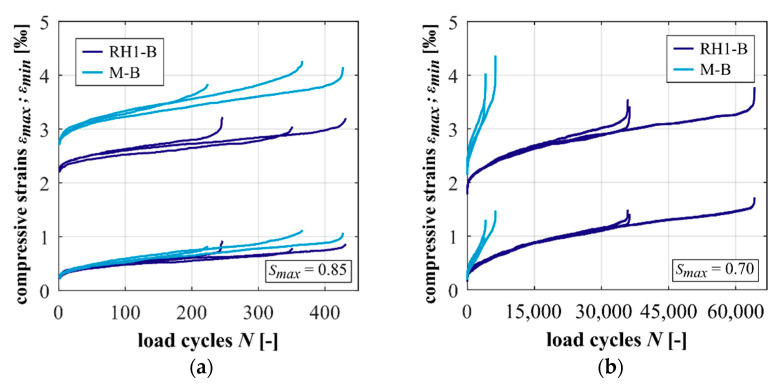
Developments of strains of the concrete and mortar at *S_max_ =* 0.85 (**a**) and 0.70 (**b**).

The developments of strain of the concrete and mortar at *S_max_ =* 0.85 and 0.70 are shown comparatively in Figure 3. The gradients of strain in phase II are flatter at the lower stress level, which corresponds to results documented in literature (e.g., [1,12]). It is obvious that the concrete has a lower initial maximum strain at both stress levels, although the respective absolute maximum stresses applied were almost identical due to the similar compressive fatigue reference strengths (cf. Section 2.2). Thus, the basalt coarse aggregate leads to a lower initial maximum strain, which corresponds to the ratio of the modulus of elasticity (cf. Table 2). From Figure 3, it can be seen that the concrete shows a lower total growth of maximum strain compared to the mortar at both stress levels. The gradients of strain in phase II are also flatter for the concrete compared to the mortar at both stress levels (cf. Table A2). This difference is significantly less pronounced at *S_max_ =* 0.85, where both materials reached similar mean numbers of cycles to failure (cf. Table A1).

The steeper gradients in phase II and the higher total growth of strain of the mortar might be traced back to the higher volume of mortar matrix in the mortar specimens, which is damaged in the fatigue process. Fatigue tests with comparable loads conducted on the basalt stone showed no indications of damage occurring (not published). Investigations on other high-strength concrete compositions emphasised the important influence of the mortar’s fatigue behaviour on the strain development of concrete [5], which superimpose with the influence of coarse aggregate.

**Figure 4:**
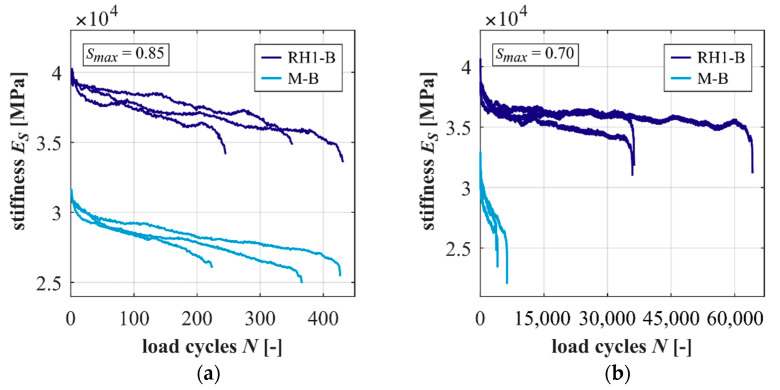
Developments of stiffness of the concrete and mortar at *S_max_ =* 0.85 (**a**) and 0.70 (**b**).

The developments of stiffness of the concrete and mortar are shown for both stress levels in Figure 4. It is obvious from Figure 4 that the concrete has a significantly higher stiffness level initially and in the complete fatigue process compared to the mortar. The gradients of stiffness of the concrete and mortar are flatter at the lower stress level, which corresponds to results from [1]. The concrete shows steeper gradients of stiffness in phase II at *S_max_ =* 0.85 and flatter gradients at *S_max_ =* 0.70 (cf. Table A2). Thus, the basalt coarse aggregate seems to increase the degradation of stiffness per load cycle in phase II at the higher stress level. At the lower stress level, the degradation of stiffness seems to be decreased. It is assumed that this effect of the coarse aggregate can be traced back to the induced inhomogeneous stress distribution, as described in [2,3]. The number of highly stressed areas at higher stress levels is larger and the magnitudes of these stresses are higher, accelerating the damage process in the mortar matrix, whereas, at lower stress levels, the stress distribution is less inhomogeneous with lower peak stresses. Here, the higher fatigue resistance of the coarse aggregate (compared to the mortar) seems to become more important for the fatigue behaviour of the concrete. The percentile reduction in stiffness also depends on the stress level. Furthermore, it is lower for the concrete compared to the mortar at both stress levels (cf. Table A2). This reveals that the amount of stiffness reduction is dependent on the concrete microstructure.

**Figure 5:**
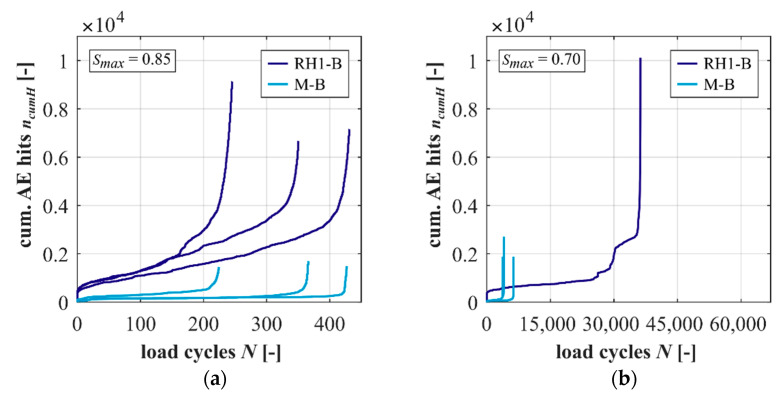
Developments of cumulated acoustic emission hits of the concrete and mortar at *S_max_ =* 0.85 (**a**) and 0.70 (**b**).

Measurements of AE hits are not available for all tests conducted with *S_max_* = 0.70 as single AE sensors detached from the test specimen surface in single tests. The developments of cumulated AE hits are shown in Figure 5 at *S_max_* = 0.85 and 0.70. When comparing Figure 5a,b, it is obvious that the concrete emits a significantly larger total number of AE hits compared to the mortar (cf. Table A2). Thus, the presence of the basalt coarse aggregate generally leads to an increased AE activity.

The concrete shows steeper gradients of cumulated AE hits in phase II at *S_max_* = 0.85. The concrete also emits more AE hits per cycle in phase II (gradient) at *S_max_* = 0.70, but the characteristic of the development is different compared to the other developments: a stepwise development after a first initial phase is observable for the concrete. The characteristics of the stepwise development with sections of high AE activity (rather vertical) followed by sections of comparably low AE activity (rather horizontal) are not visible in the macroscopic damage indicators strain and stiffness (cf. Figure 3 and Figure 4). These stepwise developments might indicate damage processes occurring on very small scales (perhaps sub-microscale) in the sections with high AE activity and, thus, are not visible macroscopically. The large number of cycles with a low number of AE hits might represent equilibrium states of these small-scale damage processes.

## 4. Computational Modelling

A finite element model was set up to predict the fatigue behaviour of different concrete mesostructures. Accordingly, the weak form of the balance of momentum is required, which reads

(3) $$\overset{}{\underset{\Omega }{\int }}\sigma :\delta \varepsilon \;d\Omega -\overset{}{\underset{\delta \Omega }{\int }}t\cdot \delta u\;d\delta \Omega +\overset{}{\underset{\Omega }{\int }}\rho \ddot{u}\cdot \delta u\;d\Omega =0\;,$$

where σ is the stress tensor, δε is the variation of the strain tensor, t is the external traction vector, δu is the variation of the displacement vector or the virtual displacement, ρ is the mass density and $\ddot{u}$ is the acceleration vector. The material model for the basalt aggregate and the mortar was chosen to be isotropic linear elastic including an isotropic damage model based on gradient-enhanced equivalent strains. An additional fatigue model was employed to reduce the remaining strength of the material depending on the deformation history. The stress tensor is computed by

(4) $$\sigma =\left(1-d\right)\sigma_{0}$$

with

(5) $$\sigma_{0}=2\mu \varepsilon +\Lambda \;tr\left(\varepsilon \right)1$$

The material parameters μ and Λ are the Lamé constants, ε is the strain tensor and d is the damage variable, which is calculated according to the damage model by Mazars and Pijaudier-Cabot [19].

(6) $$d\left(\kappa \right)=1-\frac{\kappa_{0}}{\kappa }\left(1-\alpha \right)-\alpha e^{-\beta \left(\kappa -\kappa_{0}\right)}$$

This damage model is frequently applied for concrete. Here, α and β are parameters to adjust the shape of the damage function and $\kappa_{0}$ is an initial value for the history variable κ. If κ is larger than $\kappa_{0},$ then damage starts to increase. A gradient-enhanced equivalent strain measure [20] was used for the formulation of the yield condition required to determine the internal variable κ to avoid mesh dependence of the solution. The gradient-enhanced equivalent strain measure $\bar{\varepsilon }$ is a global scalar field quantity and results from the solution of the weak form of the inhomogeneous Helmholtz equation

(7) $$\overset{}{\underset{\Omega }{\int }}\left(\bar{\varepsilon }-\tilde{\varepsilon }\left(\varepsilon \right)\right)\cdot \eta \;d\Omega +c\overset{}{\underset{\Omega }{\int }}grad\left(\bar{\varepsilon }\right)\cdot grad\left(\eta \right)\;d\Omega =0\;\;,$$

where c is the so-called characteristic length and $\tilde{\varepsilon }$ is a scalar valued function of the strain tensor ε, which can be used to account for the different damage behaviour under tension compared to the damage behaviour under compression. The so-called modified von Mises criterion is employed [21],

(8) $$\tilde{\varepsilon }\left(\varepsilon \right)=\frac{\left(k-1\right)I_{1}}{2k\left(1-2\nu \right)}+\sqrt{{\left(\frac{\left(k-1\right)I_{1}}{2k\left(1-2\nu \right)}\right)}^{2}+\frac{3J_{2}}{k{\left(1+\nu \right)}^{2}}}\;\;,$$

which was used for concrete in [22]. Here, ν is Poisson’s ratio, $I_{1}=tr\left(\varepsilon \right)$ is the first invariant of the strain tensor and

(9) $$J_{2}=\frac{1}{2}\varepsilon :\varepsilon -\frac{1}{6}{\left(tr\left(\varepsilon \right)\right)}^{2}$$

is the second invariant of the strain deviator. The parameter k can be used to weigh the different contributions of volumetric and deviatoric parts of the strain tensor. Here, k was set to k = 10 for all simulations. The damage criterion in Equation (10) was used to calculate the update of the history variable κ. This usually rather simple condition is formulated as an inequality.

(10) $$f\left(\bar{\varepsilon },\kappa \right)=\bar{\varepsilon }-\kappa \leq 0$$

If $f\left(\bar{\varepsilon },\kappa \right)<0$, then damage does not change. If the gradient-enhanced equivalent strain $\bar{\varepsilon }$ yields values such that $f\left(\bar{\varepsilon },\kappa \right)>0$, then κ needs to be adjusted such that $f\left(\bar{\varepsilon },\kappa \right)=0$ and, thus, damage increases.

Employing such a damage model in a fatigue simulation would quickly lead to a saturation of the local damage after only a few load cycles and fatigue damage would not accumulate. Recently, [9,10,11] proposed an extension of a classical phase-field model for the simulation of fracture processes. Within that model, the critical energy release rate that needs to be exceeded to obtain crack propagation was reduced according to a monotonically increasing accumulated equivalent strain measure. For fatigue simulations, this model extension can be interpreted as a reduction in the local strength of the material progressing with the number of load cycles. This model was adopted for the gradient-enhanced damage model presented here. One of the possible reduction functions for the critical energy release rate presented in [11] as a suitable function to simulate not only the initial damage processes but also the stable damage-growth phase of fatigue (phase II) is

(11) $$\phi \left(\gamma \right)=\{\begin{array}{ccc} 1 & : & \gamma <\gamma_{t}\\ {\left(1-a\;\log\left(\gamma /\gamma_{t}\right)\right)}^{2} & : & \gamma_{t}\leq \gamma \leq 10^{1/a}\gamma_{t}\\ 0 & : & \gamma >10^{1/a}\gamma_{t} \end{array}$$

The parameter a can be regarded as a parameter to set the strain history-dependent rate of strength reduction in the material. The parameter $\gamma_{t}$ is a threshold value for γ beyond which the strength of the material is reduced. The scalar valued field variable γ describes the accumulated history of the strain and is only allowed to grow monotonically, i.e., $\dot{\gamma }\geq 0$. There are multiple possibilities of the definition of this variable. Some of them are described in [11] in the case of a phase-field formulation. Here, γ is set to

(12) $$\gamma =\overset{t}{\underset{0}{\int }}H\left(\bar{\varepsilon }\dot{\bar{\varepsilon }}\right)\left|\dot{\bar{\varepsilon }}\right|\;d\tau$$

where H is the Heaviside function. By this choice, regarding cyclic loading, only the loading part contributes to the increase in γ and not the unloading part, which corresponds to experimental results documented in literature (e.g., [23]). Furthermore, Equation (12) guarantees a monotonic decrease in the strength of the material. The reduction function ϕ(γ) (Equation (11)) now still needs to be included in the yield function for the history variable κ.

(13) $$f\left(\bar{\varepsilon },\kappa ,\gamma \right)=\bar{\varepsilon }-\phi \left(\gamma \right)\kappa \leq 0$$

This eventually means that the threshold value for $\bar{\varepsilon }$, beyond which damage increases, decreases cycle by cycle. This is not identical to the approach of [11], where the critical energy release rate value is reduced by the function ϕ(γ). However, simulation results show that the same effect can be achieved by introducing the modified yield function $f\left(\bar{\varepsilon },\kappa ,\gamma \right)$. It needs to be emphasised that this model does not rely on phenomenological assumptions based on the Paris law. Quite contrary to that, it is possible to predict the damage growth during the initial damage phase, the Paris regime and the unstable damage growth phase by applying the model.

## 5. Comparison between Experimental and Computational Results

In the following, the results of the numerical simulations of the concrete and the mortar are compared with the experimentally determined strain developments at the maximum stress level of *S_max_* = 0.85 and minimum stress level of *S_min_* = 0.05. Since the number of cycles to failure for those stress levels is rather low, it is not necessary to apply computational techniques, such as cycle jump methods, here, which avoid excessive computational effort in case of large numbers of cycles. A standard Newmark method is applied as a time integration method for the following simulations. Twenty time steps are simulated for each cycle. The parameters of the simulation of the mortar and those of the simulation of the concrete are given in Table 3.

### 5.1. Simulation of the Mortar

**Figure 6:**
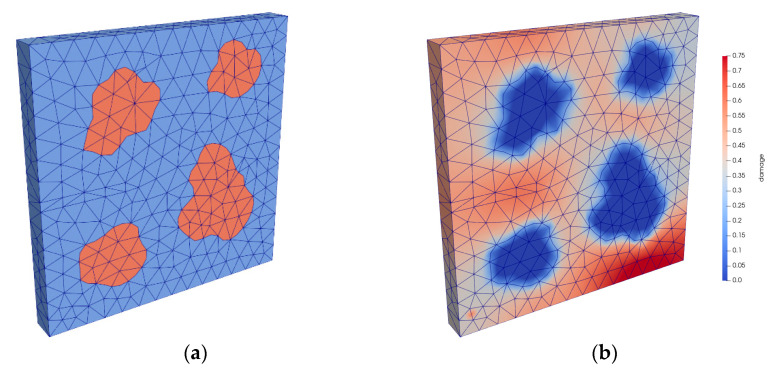
Discretisation of the mortar (**a**) and computed gradient-enhanced damage distribution at *N/N_f_* = 0.99 (**b**).

A 3 mm × 3 mm × 0.3 mm material sample was investigated for the simulation of the mortar (cf. Figure 6a). The domain consisted of basalt grains with a diameter *d* < 2 mm and a homogenised mortar matrix consisting of cement paste and other components. The dimensions of the material sample considered were chosen to be small to keep the simulation times for this initial simulation reasonably low.

The domain was discretised using tetrahedral elements with quadratic shape functions. The boundary conditions were set such that the bottom surface could not move in vertical direction, the back surface could not move to the front and the force-controlled load was applied to a rigid diaphragm on the top surface. The basalt grains were considered to behave linearly elastically without exhibiting damage. This corresponds to our own fatigue tests conducted on the basalt stone. The material parameters of the mortar matrix and the basalt grains are given in Table 3. The computed gradient-enhanced damage distribution is shown for *S_max_* = 0.85 after 340 load cycles, or rather, *N/N_f_* = 0.99, i.e., shortly before failure occurs, in Figure 6b.

**Figure 7:**
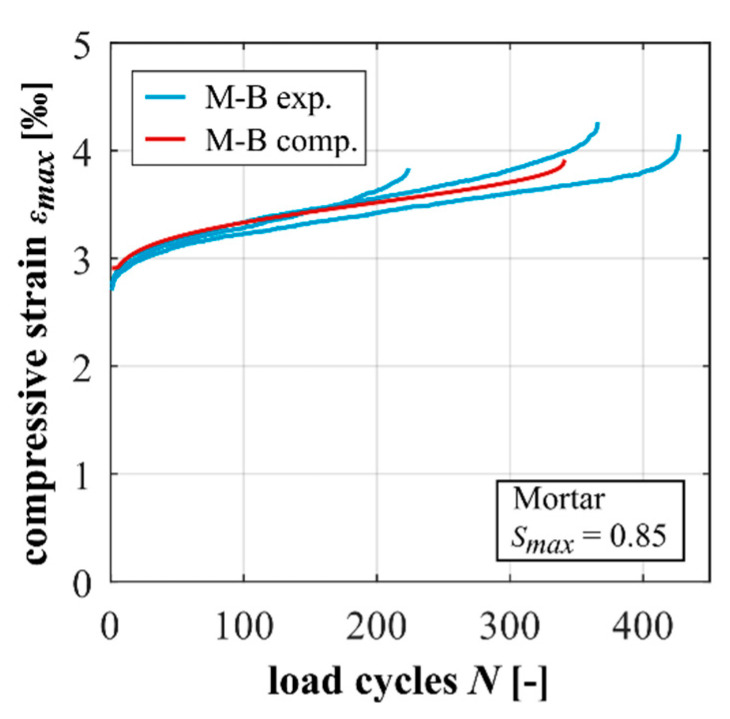
Experimentally and computationally determined strain developments of the mortar.

The experimentally and computationally determined developments of maximum strain are shown in Figure 7. The same as before in Section 3.2, the experimentally determined developments are displayed as single curves. It can be seen that the computational result is in very good agreement with the experimental results. The computed number of cycles to failure, comprehended as a computed mean value, corresponds to the numbers of cycles to failure tested. Furthermore, the gradient in phase II and the characteristics of phase I and phase III are very well depicted, which is remarkable.

### 5.2. Simulation of the Concrete

**Figure 8:**
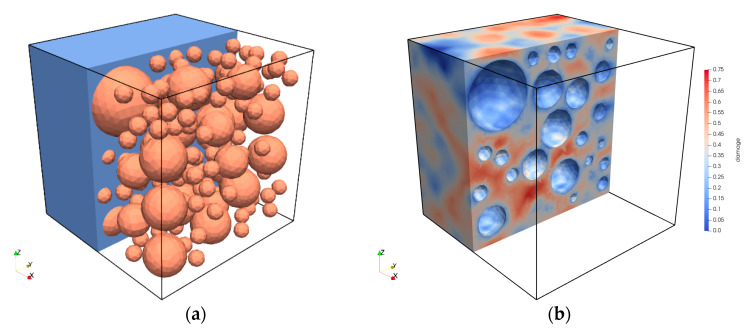
Discretisation of the concrete (**a**) and computed gradient-enhanced damage distribution at *N/N_f_* = 0.99 (**b**).

A cube-shaped material sample with an edge length of 23 mm was modelled for the simulation of the concrete (Figure 8a). The randomly distributed basalt grains were assumed to have spherical shapes. Their diameter followed the real grain size distribution in the range of grain diameters *d* ≥ 2 mm, which was experimentally determined for the basalt aggregate used. The grain size distribution used for discretisation is shown in Table A3 in the appendix. Here, the homogenised mortar matrix consisted of cement paste, basalt grains with diameters *d* < 2 mm and other components.

The same as for the mortar, the domain was discretised using tetrahedral elements with quadratic shape functions. Symmetry boundary conditions were applied in all directions. Again, the force-controlled load was applied to a rigid diaphragm on the top surface. The basalt grains were considered to behave linearly elastically again. The material parameters for the concrete simulation are given in Table 3.

The computed gradient-enhanced damage distribution is shown for *S_max_* = 0.85 after 306 load cycles, or rather, *N/N_f_* = 0.99, in Figure 8b. It can be seen that damage accumulates in the mortar matrix depending on the size and relative position, or rather, distance of the grains to each other and on the load direction. This result generally corresponds to the results and suggestions of [2,3,5].

**Figure 9:**
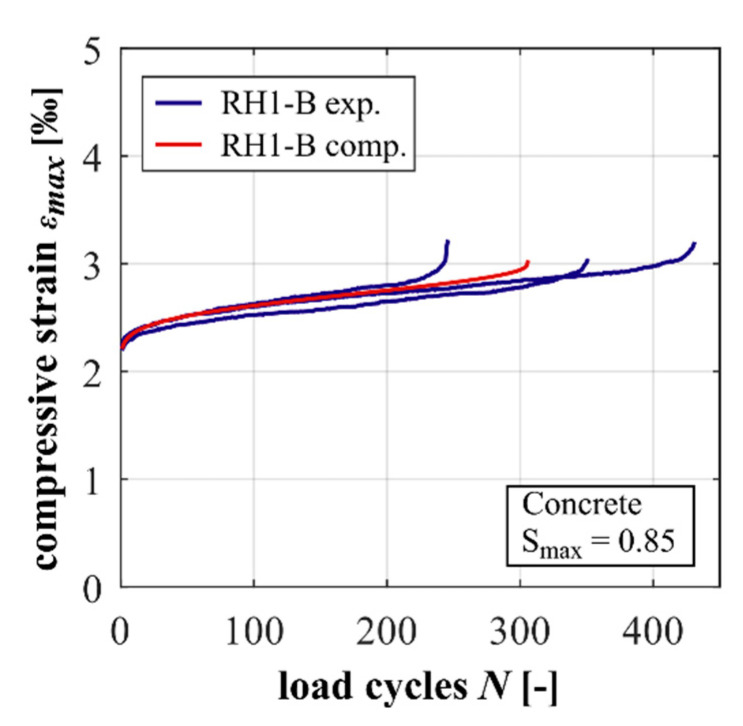
Experimentally and computationally determined strain developments of the concrete.

The experimentally and computationally determined developments of maximum strain are displayed in Figure 9. Again, the experimentally determined results are displayed as single curves. Similar to the results for the mortar, the comparison of the computational results and the experimental measurements shows a good agreement. Both the gradient in phase II and the characteristic of phase I and III are very well-depicted. The calculated number of cycles to failure also shows a good correlation with the experimental results in terms of a mean value.

## 6. Summary and Conclusions

The results from experimental and numerical investigations on a high-strength concrete and its corresponding mortar subjected to compressive fatigue loading with two stress levels are presented in this paper. The concrete reached similar numbers of cycles to failure as the mortar at the higher stress level but higher numbers of cycles to failure at the lower stress level. Comparing the results for the concrete and mortar, the total growth of strain is smaller and the gradient in phase II is flatter for the concrete. However, the gradient of stiffness of the concrete is steeper at the higher stress level but flatter at the lower stress level, indicating a negative effect of the basalt coarse aggregate on the reduction in stiffness at the higher stress level. Furthermore, larger AE activity was determined for the concrete compared to the mortar and, thus, must be traced back to the presence of coarse aggregate. A stepwise increased AE activity in the concrete at the lower stress level indicates damage processes on a very small scale (perhaps on a sub-microscale), which are not visible in the macroscopic damage indicators. The characteristics of the AE signals will be analysed in more detail in further analyses in order to obtain more knowledge about the damage processes.

The experimental results presented in this paper demonstrate the existence of differences between the fatigue behaviour of concretes and mortars due to the presence of coarse aggregate. It is assumed that the highly fatigue-resistant coarse aggregate in the concrete relieves the stresses in the fatigue-sensitive mortar at lower stress levels. However, at the higher stress level, indication exists that the coarse aggregate has a negative effect on the concrete’s fatigue behaviour (higher stiffness degradation) compared to that of the mortar. This might be traced back to the inhomogeneous stress distribution induced due to the higher stresses applied and the higher resulting differences in the deformation behaviour of the coarse aggregate basalt and the mortar. Despite this, similar numbers of cycles to failure are reached. However, the influence of coarse aggregate and mortar matrix superimpose. The results in [5] show that the ratio of the volume of mortar and aggregates in concrete specimens together with the damage sensitivity of the mortar, ratio of stiffness and the fatigue resistance of the aggregates lead to a certain fatigue behaviour of the concrete. This superimposition leads to less deviating strain developments and similar numbers of cycles to failure at the higher stress level, which seems to be a rather random result.

A newly developed model for the simulation of the strain development under compressive fatigue loading is presented in this paper. The comparison of the experimentally and computationally determined developments of strain reveal very good agreement. The numbers of cycles to failure and the characteristics of the strain developments are very well-depicted. Furthermore, the simulated numbers of cycles to failure result from the simulated fatigue damage development in the mesostructure, whereby the correlation of the slope of the strain development with the number of cycles to failure reached fits the experimental results very well. In contrast to damage models based on the assumption of the Paris law, the newly introduced computational model allows for the simulation of phases I, II and III of the fatigue damage process. A significant and remarkable advantage of the proposed model compared to most classical gradient-enhanced damage approaches is that it can be applied to simulations with monotonically increasing loading and fatigue simulations. The stress redistribution in the mesostructure due to damage can be visualised by applying the fatigue damage model presented. This can contribute to a better understanding of the damage processes within the concrete mesostructure.

The present model does not yet contain elastoplastic or viscoplastic material behaviour, which have a significant effect on the fatigue behaviour of concrete. Nevertheless, the results obtained are promising and the material model can be extended by plasticity models suitable for concrete, which will be the subject of future collaborative work. In addition to that, efficient computational techniques avoiding excessive computational effort can be applied for the simulation of large numbers of cycles at lower stress levels in the future.

## Figures and Tables

**Table 1:** Composition of the concrete RH1-B investigated.

Component	Content
Portland Cement (CEM I 52,5 R HS/NA)	500 kg/m^3^
Quartz sand (0/0.5 mm)	75 kg/m^3^
Sand (0/2 mm)	850 kg/m^3^
Basalt (2/5 mm)	350 kg/m^3^
Basalt (5/8 mm)	570 kg/m^3^
Superplasticiser	5.00 kg/m^3^
Stabiliser	2.85 kg/m^3^
Water	176 kg/m^3^

**Table 2:** Mean values of the 28-day compressive strength and modulus of elasticity.

Composition	RH1-B	M-B
*f_cm,cube_* (MPa)	113	108
*E_cm_* (MPa)	40,000	34,900

**Table 3:** Material parameters for the computational simulation of the mortar and concrete.

Material Parameter	Basalt	Mortar Matrix (Mortar Sim.)	Mortar Matrix (Concrete Sim.)
shear modulus μ (MPa)	38,682	14,536	14,536
Lamé constant Λ (MPa)	53,418	9691	9691
mass density ρ (g/cm^3^)	2.97	2.27	2.27
length scale parameter c (mm^2^)		0.1	0.3
$\mathrm{damage}\;\mathrm{parameter}\;\kappa_{0}$ (-)		0.000150	0.000155
damage parameter α (-)		0.7	0.7
damage parameter β (-)		1950	2300
fatigue parameter a (-)		0.028	0.022
$\mathrm{fatigue}\;\mathrm{parameter}\;\gamma_{t}$ (-)		0.0015	0.00015

## Data Availability

Data available on request.

## Appendix A

**Table A1:** Single and mean numbers of cycles to failure *N_f_*.

	*S_max_* = 0.85		*S_max_* = 0.70	
	M-B	RH1-B	M-B	RH1-B
single *N_f_*	427	431	4048	64,209
	224	351	6301	35,871
	366	246	10,566	36,267
mean *N_f_*	339	343	6972	45,449

**Table A2:** Mean values of parameters of damage indicators of the mortar and concrete.

		*S_max_ =* 0.85		*S_max_ =* 0.70	
Parameter	Unit	M-B	RH1-B	M-B	RH1-B
$\Delta \varepsilon_{\max}^{0.0–1.0}$	(‰)	1.34	0.94	1.99	1.77
$\Delta \varepsilon_{\min}^{0.0–1.0}$	(‰)	0.75	0.64	1.21	1.39
${\mathrm{grad}\;\varepsilon }_{\max}^{0.2–0.8}$	(-)	2.41 × 10^−6^	1.40 × 10^−6^	16.60 × 10^−8^	1.90 × 10^−8^
${\mathrm{grad}\;\varepsilon }_{\min}^{0.2–0.8}$	(-)	1.43 × 10^−6^	0.80 × 10^−6^	11.60 × 10^−8^	1.70 × 10^−8^
$\Delta E_{S}^{0.0–1.0}$	(%)	19.03	14.60	27.69	20.08
${\mathrm{grad}\;E}_{S}^{0.2–0.8}$	(MPa)	−9.03	−10.82	−0.60	−0.03
*Σn_H_*	(-)	1543	6854	2318	10,086
${\mathrm{grad}\;n}_{\mathrm{cumH}}^{0.2–0.8}$	(-)	0.71	9.05	0.014	– (*)

(*) not determinable.

**Table A3:** Grain size distribution of basalt coarse aggregate for the discretisation of concrete.

Diameter of Particles	Number of Particles in Specimen
2 mm	165
4 mm	16
5 mm	29
8 mm	2
